# Is Biblioleaks Inevitable?

**DOI:** 10.2196/jmir.3331

**Published:** 2014-04-22

**Authors:** Adam G Dunn, Enrico Coiera, Kenneth D Mandl

**Affiliations:** ^1^Centre for Health InformaticsAustralian Institute of Health InnovationThe University of New South WalesSydneyAustralia; ^2^Center for Biomedical InformaticsHarvard Medical SchoolBoston, MAUnited States; ^3^Children’s Hospital Informatics ProgramHarvard-MIT Health Sciences and TechnologyBoston Children’s HospitalBoston, MAUnited States

**Keywords:** bibliographic databases, compromising of data, open access, public access to information, peer-to-peer architectures

## Abstract

In 2014, the vast majority of published biomedical research is still hidden behind paywalls rather than open access. For more than a decade, similar restrictions over other digitally available content have engendered illegal activity. Music file sharing became rampant in the late 1990s as communities formed around new ways to share. The frequency and scale of cyber-attacks against commercial and government interests has increased dramatically. Massive troves of classified government documents have become public through the actions of a few. Yet we have not seen significant growth in the illegal sharing of peer-reviewed academic articles. Should we truly expect that biomedical publishing is somehow at less risk than other content-generating industries? What of the larger threat—a “Biblioleaks” event—a database breach and public leak of the substantial archives of biomedical literature? As the expectation that all research should be available to everyone becomes the norm for a younger generation of researchers and the broader community, the motivations for such a leak are likely to grow. We explore the feasibility and consequences of a Biblioleaks event for researchers, journals, publishers, and the broader communities of doctors and the patients they serve.

## The Hypothetical Biblioleaks Scenario

Through a concerted effort, hackers gain access to the databases of six publishers that together control access to the majority of subscription-based biomedical journal articles. This group makes copies of every article from every journal and releases them into the public domain. Subsets of articles are mirrored in anonymous peer-to-peer networks, creating a decentralized and multiply-redundant repository that is accessible to any human or computer algorithm. The repository grows when its users begin to add new and missing articles, creating a self-sustaining system of frictionless, free, and universal access to published research. While there would be recourse against offenders and while the wider academic community may be unlikely to embrace illicit activity, a robust international article-sharing underground is created. Academics in wealthy countries generally enjoy the privilege of institutional subscriptions to many journals, but articles that require payment to read or download (paywalled) are largely beyond the reach of everyone else and there is a substantial motivation to access this new resource.

The potential for this form of guerrilla open access is rarely discussed [1], despite the massive scale of recent cyber-attacks against commercial and government interests. Large-scale events, like the rise of illegal music file sharing on Napster and the massive releases of government documents including The War Logs and global surveillance disclosures, can force these issues of access and transparency into the mainstream public debate.

The likely consequences of such an event for publishers, journals, researchers, and the wider community are largely unknown. Speculating on the consequences of a leak and the plausibility of a decentralized article-sharing underground, we consider the current behaviors of the producers and consumers of biomedical research, the sizes and forms of recent data breaches, and the technologies underpinning anonymous sharing.

## Current Public Access

**Figure 1:**
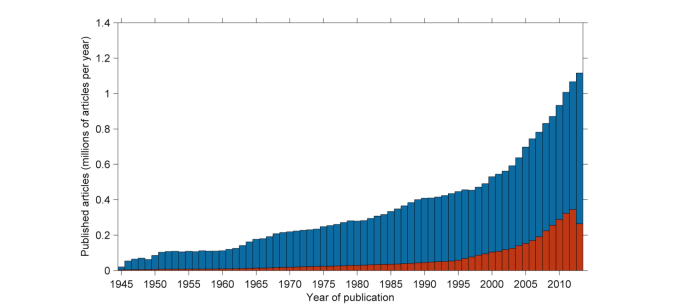
Volumes of articles currently indexed by PubMed (blue) and volumes freely available via PubMed Central (orange), arranged by year of publication, for articles published between 1945 and 2013 (data accessed 17 March 2014).

Of the 23.6 million articles currently indexed by PubMed (a search engine that accesses the MEDLINE database of life science and biomedical literature), the full text versions of just over 3 million are available for free via PubMed Central (Figure 1). This means that today around 13% of peer-reviewed biomedical articles currently indexed by PubMed are directly available for free via PubMed Central. Although open access publishing is growing rapidly (a 16-fold growth between 2000 and 2011 [2]), the overall volume of publishing in biomedical research appears to be outpacing the volume of growth in open access, creating a persistent archive of potentially inaccessible biomedical research.

PubMed indexes the bulk of all biomedical research that meets a minimum standard of quality but PubMed Central does not capture all of the articles that can be accessed for free. Other access options include library or personal journal subscriptions, emailing authors, a series of balkanized repositories like research-based social networks and institutional webpages [3], and paying publishers for access to individual articles. This process for gaining access to the full text of paywalled articles is inefficient even for experts actively engaged in research, but its most severe effects are likely felt by the groups that have fewer options for access—clinicians and the broader public.

## Feasibility

The motivation behind the ethos that “all information should be free” has been explicitly built into the fabric of academia for at least 50 years [4]. Where the costs of accessing published research are unaffordable, the drivers to subvert access restriction seem no different to those driving recent large scale data breaches. In a time where once secret or restricted information is increasingly leaked in the public interest, and at least one advocate has publicly promoted guerrilla open access in peer-reviewed literature [1], publishers should address the threat of a massive data breach.

**Figure 2:**
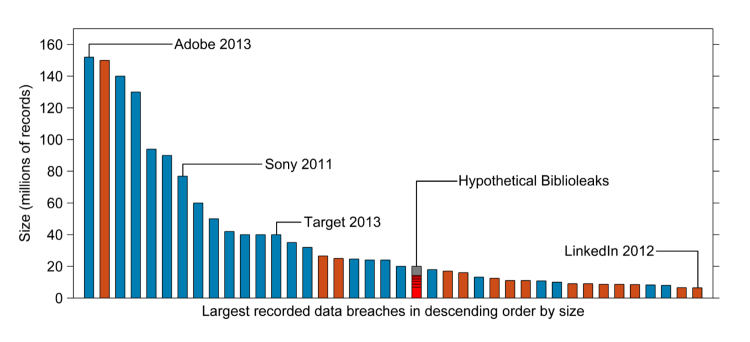
Largest recorded data breaches by number of records (accessed 7 January 2014). Hacks are in blue, all other breach types in orange (eg, stolen/lost disks)–compared to a hypothetical breach equivalent to the numbers of articles indexed by PubMed for which full-text versions require a subscription or payment to access. The proportions associated with the 6 largest publishers (sampled from outgoing PubMed links on 7 January 2014) make up 72% of these inaccessible articles (in red).

Scale is no barrier to a cyber-attack. From a database of over 7600 recorded data breaches [5], 21 involved over 20 million records each, indicating clear and recent precedents for a data breach of this scale (Figure 2). Among the 21 largest, hackers were responsible for 18, and most of these were in the last five years. From these records, it is clear that large businesses are not immune to data breaches and that large data breaches are increasing unabated.

Once released into the public domain, articles may be difficult or impossible to recover because there are no technical barriers to leaking published research once it has been acquired. The software used for cleaning documents and anonymously disseminating them online are available [6,7]. The peer-to-peer network structures that could be used to store, track, and provide access to the leaked articles became mainstream with Napster in the late 1990s [8,9]. While publishers are currently involved in issuing take-down notices to authors and institutions that release their own articles in contravention of licenses, this strategy for enforcing copyright ownership could not be used if articles were leaked anonymously online. New forms of peer-to-peer networks also resist this form of censorship through the privacy and security of darknet structures [10,11], and by using distributed storage, where files are split into encrypted chunks so that all users have access but no individual user stores an entire file [11].

## Publishers

The publishing industry has experimented with a variety of open access models [12,13]. Traditional publishers have embraced gold, green, and hybrid open access models for both new and existing journals. In gold open access, authors typically pay to publish at the time of publication and articles are then free to access. This does not necessarily mean that the articles are released without restrictions on their use, however. In green open access, authors are permitted to upload some version of the article to a repository, sometimes after a delay. Traditional publishers continue to be exceedingly profitable even when the majority of their articles are released under green open access licenses [14], and new predatory publishers have also flourished in an ever-expanding market [15,16].

The commercial risk of a massive data leak would be skewed toward traditional publishers with business models involving charging for access to published research, and journals that rely on traffic through their websites for advertising revenue. Such a scenario may also affect the community structure in some disciplines by redirecting readership away from journal websites, reducing interaction within societies [17]. In a Biblioleaks scenario, open access publishers that receive the bulk of their income at the point of publication and do not rely on subscriptions or advertising revenue would therefore be at an advantage relative to other publishers.

## Authors

The license given by subscription journals to authors typically allows them to share their published work by uploading versions of their manuscripts to government and academic institutional repositories, or personal webpages. Known as green open access, around 81% of published articles fall into to this category (62% immediately after publication, the rest after varying delays) [2]. Despite the growing requirement from public research funding bodies that research be open access, only around 12% of green open access articles can be found by searching repositories or personal webpages because authors have not availed themselves of this option [2]. What this means is that despite the ability and obligation to do so, the rate of self-archiving by researchers is low.

In contrast to the extraordinary expansion of the Napster music sharing network in the late 1990s, relatively few researchers are involved in guerrilla open access—sharing articles in contravention of the conditions of a publisher. Two sharing practices have recently emerged on Twitter. The first, #icanhazpdf [18], started in 2011 and is a “pull” practice, where those who need articles request them and wait for someone with access to provide a copy. The second, #pdftribute [19], started in 2013 as a “push” practice, where authors advertised an online location for full versions of articles they wrote or held. Both practices stabilized at a low rate of requests and uploads. From this evidence, and given the low levels of observed self-archiving and civil disobedience from within the academic community, we speculate that a disruptive change is more likely to come from a Biblioleaks scenario—a small number of massive breaches, potentially from outside academia, rather than *en masse* civil disobedience from within academic communities.

A massive leak would appear to have few or no direct negative consequences for individual researchers. The major impact would be increased access to their published works. Overall, the capacity to better link and synthesize research could also lead to improvements in automated literature discovery [20,21], expanding opportunities for new forms of research. With passive roles in the Biblioleaks scenario, the interests of authors of peer-reviewed research would be served, with no directly associated risks.

## Clinicians and Patients

While access to published literature is problematic for researchers inside universities, the broader community faces formidable barriers. There is some evidence to suggest that the public want access to published research and are hampered by paywalls [22]. Evidence showing that open access articles are viewed and downloaded more often without necessarily leading to higher citation rates is a further hint that the wider community is engaging with peer-reviewed research [23].

In the medical context, we know that health care workers are less likely to read peer-reviewed literature than they are to ask colleagues, use reference books, or visit websites via Google or Wikipedia [24]. Among patients in the United States, 58% looked online for health information and one in four encountered a paywall [25]. The evidence suggests that clinicians and the public try to use the Internet to find literature but are often unable to reach what they need. With no paid institutional access, and without the personal networks to help circumvent access restrictions, the average member of the community is more likely to ignore inaccessible articles and rely on sources that are not peer-reviewed. As such, a massive leak has every chance of creating a more informed clinical and patient community once they become comfortable with accessing such a repository.

At the population level, the gap between research consensus and public understanding has major consequences for global health, where for issues including vaccination, homoeopathy, and climate change, there is a clear dissonance between what peer-reviewed evidence shows and what large sections of the public believe [26-28]. While prior beliefs feature heavily in decision making, the first document accessed in a search plays an important role in the potential to switch beliefs [29]. By removing the barriers that restrict access to most peer-reviewed literature, a massive leak could help to reduce problematic public opinions by providing greater transparency and shifting the weight of available information away from grey literature and toward peer-reviewed research.

## Futures

Even as open access increases, the motivations for a massive leak will persist because the archive of inaccessible research continues to grow. The threats are clear. Chelsea Manning and Edward Snowden demonstrated that individuals can bring about tectonic shifts in the ability of government to maintain secrecy and the public attitudes toward clandestine programs, although at great personal cost to the leakers themselves. The entertainment industry navigated similar terrain over a decade ago, when consumers moved from recording onto cassettes from the radio to sharing on local and then global computer networks. That disruption left a legacy of file-sharing networks that have become increasingly secure and resistant to censorship. Today, instantaneous access to music, television, and movies is taken for granted by many, while the entertainment industry continues to flourish by finding legitimate and low-cost ways to reach audiences that would otherwise turn to illegal file-sharing services.

From this view, biomedical publishing faces threats, but also opportunities. The current forms of illegitimate sharing in academia rely largely on personal networks or easily censored websites. Relatively few academics have started to explore broader forms of civil disobedience. Since open access has become a mainstream issue, academics and the public are beginning to expect free and immediate access to new research as the norm and not the exception. As forms of illegitimate sharing become more sophisticated and widespread, publishers face a situation reminiscent of the one faced by the entertainment industry more than a decade ago.

We think that low-level civil disobedience (or authors unaware of which versions of their articles they are allowed to upload to repositories) is by itself unlikely to lead to a critical mass of illegal article sharing. Large-scale leaks are a bigger threat because they could immediately influence the way published research is accessed. For this reason, publishers might see value in strengthening the systems already in place to detect and prevent unusually large volume downloads, or atypical systematic or ordered access to full texts.

Prescient publishers may also consider alternatives that would minimize the motivation behind any illegal access and avoid the costs of a technical and legal arms race that may only delay the inevitable. Publishers may choose to deliberately release articles on their own terms, an approach that improved the reputation of and trust in GlaxoSmithKline when they responded to growing demand for access to comprehensive clinical trial data [30]. They might also consider alternative forms of low-cost access that could greatly expand the market for peer-reviewed research into the broader community. Examples of new forms of low-cost access, such as time-limited rentals, are already available [31].

From the limited evidence available in this area, it seems clear that a Biblioleaks event is technically feasible. There is some evidence that new forms of illegal file sharing are emerging among researchers and the broader community, suggesting that the current environment is similar to the nascent period of illegal file sharing. In that time, online users increasingly encountered the tools that provided free access to music, and fragmented communities began to coalesce into a global sharing network. If precipitated by targeted data breaches, a similar growth in underground article sharing could see negative effects for some publishers, disruptive changes to the way biomedical research is accessed by the public, the rapid development of new low-cost access options, and improved public engagement with medical research.
